# High-efficiency bio-inspired hybrid multi-generation photovoltaic leaf

**DOI:** 10.1038/s41467-023-38984-7

**Published:** 2023-06-08

**Authors:** Gan Huang, Jingyuan Xu, Christos N. Markides

**Affiliations:** Clean Energy Processes (CEP) Laboratory, Department of Chemical Engineering, Imperial College London, London, UK

**Keywords:** Solar energy, Mechanical engineering

## Abstract

Most solar energy incident (>70%) upon commercial photovoltaic panels is dissipated as heat, increasing their operating temperature, and leading to significant deterioration in electrical performance. The solar utilisation efficiency of commercial photovoltaic panels is typically below 25%. Here, we demonstrate a hybrid multi-generation photovoltaic leaf concept that employs a biomimetic transpiration structure made of eco-friendly, low-cost and widely-available materials for effective passive thermal management and multi-generation. We demonstrate experimentally that bio-inspired transpiration can remove ~590 W/m^2^ of heat from a photovoltaic cell, reducing the cell temperature by ~26 °C under an irradiance of 1000 W/m^2^, and resulting in a relatively 13.6% increase in electrical efficiency. Furthermore, the photovoltaic leaf is capable of synergistically utilising the recovered heat to co-generate additional thermal energy and freshwater simultaneously within the same component, significantly elevating the overall solar utilisation efficiency from 13.2% to over 74.5%, along with over 1.1 L/h/m^2^ of clean water.

## Abstract (web summary)

Most sunlight received by photovoltaic panels is converted to and lost as heat, increasing their temperature and deteriorating their performance. Here, the authors propose a multi-energy generation photovoltaic leaf concept with biomimetic transpiration and demonstrate much improved performance.

## Introduction

By far the highest growth and new investment in renewable energy technologies globally are being experienced by the solar sector, and especially photovoltaic (PV) systems that have experienced an average growth rate of close to 24% per year in the past 5 years (2017–2021)^1^. Global PV capacity crossed 700 GW in 2020^1^ and is estimated to reach ~22 TW in 2050^2^, as part of plans to attain a carbon-free power supply by 2050.

PV cells are usually sensitive to a portion of the solar spectrum (e.g. 300–1100 nm for single-junction Si cells), with only 10–25% of the incident solar energy converted into electricity by commercial PV panels^3–6^. The rest of the electrically unusable solar energy, which accounts for over 70% of the incident energy, dissipates as waste heat in PV cells^4^, and results in an increase in their operating temperature. As a consequence, the temperature of PV cells can exceed 65 °C^7–9^ in hot and sunny conditions, leading to a significant decrease in electrical efficiency^10^. The efficiency of most common Si-based PV panels typically decreases by 4.0–6.5%^11^ and their ageing rate doubles for every 10 °C increase in operating temperature^12^. Therefore, significant potential to generate additional electricity from the over 700 GW existing PV installations globally is currently lost due to the lack of thermal management, and this loss will worsen as global PV capacity continues its explosive growth. High-efficiency and low-cost thermal management approaches for PV panels are of great significance in this context, as these would allow significantly enhanced power generation of dozens of GW from current global PV installations, and with a potential to mitigate the loss of hundreds of GW in future installations.

Active thermal management methods have proven to be effective at removing heat from PV panels by employing either water or air flows^13–16^, however, they usually require heat exchanging structures (e.g. fin structures, metal thermal absorbers, etc.) and/or hydraulic structures (e.g. piping and pumps), which introduces design, installation and operational complexity as well as additional cost, and are associated with parasitic electricity consumption to drive the coolant flows^17^. Passive thermal management methods, the most common of which rely on natural convection cooling, on the other hand, usually have simpler structures but are also limited to lower cooling rates. Another passive thermal management method is based on sub-bandgap reflection, and is capable of cooling Si cells by ~4 °C^18^. Recently, advanced passive thermal management methods for PV panels have been attracting increased attention. For example, passive radiative thermal management methods employ selectively-emissive coatings in order to emit heat via radiation to the cold outer space^19,20^. These methods have a typical cooling power of only 40–140 W/m^2^ under a cloudless sky^21,22^, which is lower than the ~200–400 W/m^2^ that can be achieved by natural convection, mentioned above. The combination of radiative cooling with the sub-bandgap reflection method can further improve the cooling performance^23^. An impressive passive cooling power of ~300 W/m^2^ has been achieved by utilising atmospheric water, decreasing the operating temperature by ~10 °C under 1000 W/m^2^ solar irradiance^24^.

Despite recent progress, there remains significant room for more efficient thermal management solutions aimed at PV-based systems, with substantial added benefits if this can be done by synergistically utilising the recovered heat for multi-generation purposes, i.e. for delivering one or more additional useful energy vectors, instead of rejecting it to the surroundings. This remains a crucial challenge for existing PV thermal management technologies, but also presents a great opportunity for radically boosting the solar utilisation efficiency of PV-based solar technologies.

Here, we aim to co-generate electricity, heat and clean water from the same pumpless ‘hybrid’ solar collector, while performing thermal management to improve PV electrical performance. This is done by implementing a highly effective thermal management method inspired by the transpiration of plant leaves. Trees are extremely effective in moving water from the soil to a height of tens of (and in some cases more than one hundred) metres for transpiration, in order to keep their leaves always cool and to protect their most important functions, i.e. photosynthesis process^25,26^. These natural solutions are particularly promising, as they are capable of maintaining leaf temperatures within a stable range, regardless of the weather^27^. Transpiration also plays important roles in the water cycle and in freshwater generation in nature, especially in rainforest areas. In this study, we propose a bio-inspired hybrid multi-generation photovoltaic-leaf (PV-leaf) with: (i) a biomimetic transpiration structure, featuring a specific design and materials selection (bamboo fibres and stacked hydrogel cells), to drive water flows passively from a separate water tank to the solar cell without a pump; while (ii) covering the whole area of the cell with water and evaporating the water with high effectiveness within the collector to capture clean water vapour and heat simultaneously with high efficiency in addition to electricity from the same component.

The transpiration performance of the PV-leaf is demonstrated experimentally as being capable of removing 75% (590 W/m^2^) of the heat in the PV cell, significantly decreasing the PV cell’s operating temperature by ~26 °C relative to a standalone PV cell. The PV-leaf is shown to have a capability of passive control, adapting to different ambient temperatures and also a strong compatibility for utilising different working fluids (e.g. water and saline). Importantly, the PV-leaf is capable of synergistically utilising the PV heat to produce additional fresh water and thermal energy, significantly elevating the overall solar utilisation efficiency from 13.2% to 74.5% (~5–6 times that of a standalone PV cell). This paper presents the hybrid multi-generation PV-leaf concept, includes details concerning the design of a proof-of-concept PV-leaf including of the biomimetic transpiration structure, and proceeds to report results from indoor and outdoor tests both for the transpiration performance under various working conditions, and of the multi-generation performance of the PV-leaf for combined electricity, thermal energy and clean water provision. The biomimetic transpiration structure is constructed from affordable, readily-available and environmentally-friendly materials, allowing these systems to be mass manufactured and to compete economically and environmentally with established technologies.

## Results

### PV-leaf configuration and working principle

**Fig. 1:**
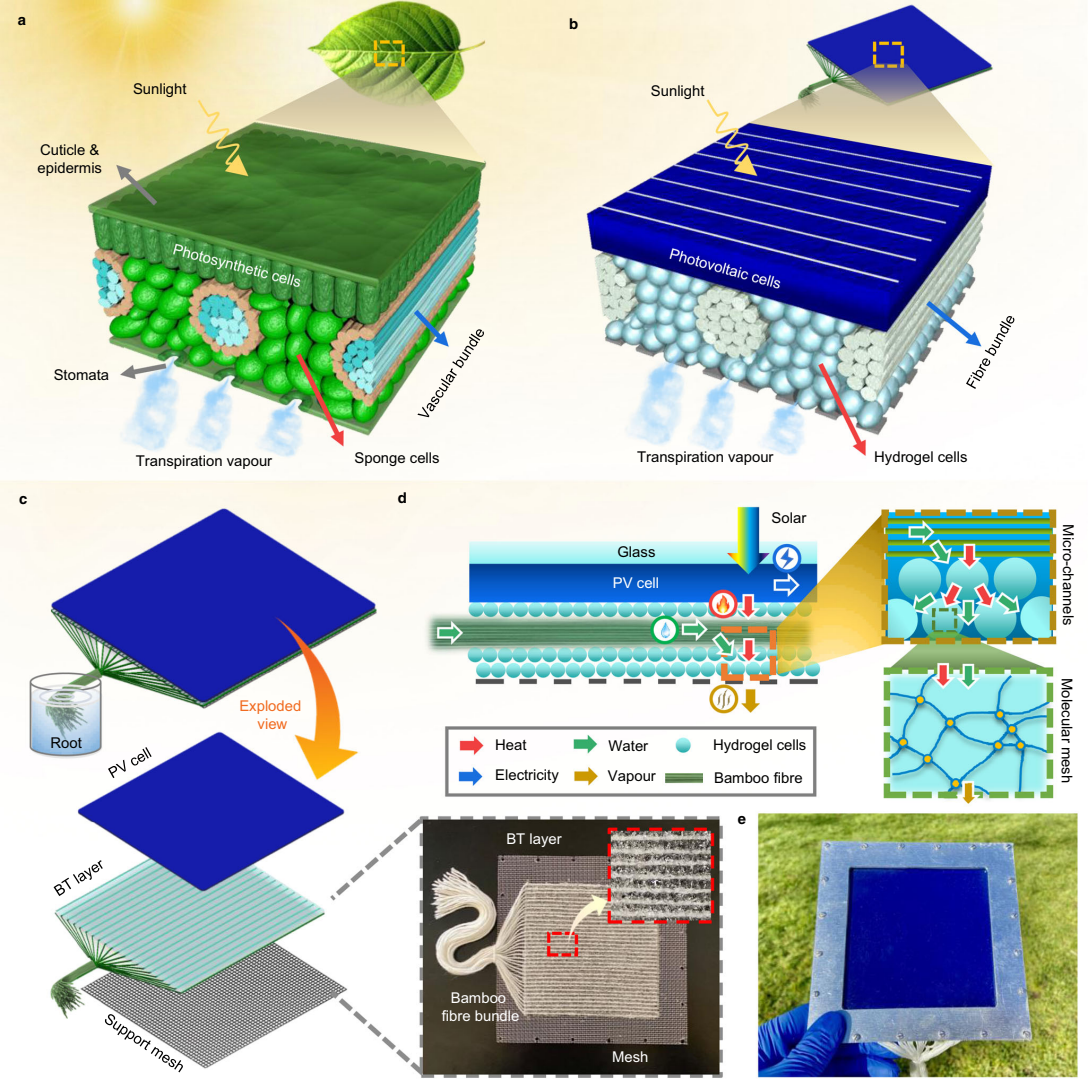
Schematic illustration of the PV cell and transpiration structure arrangement within the bio-inspired PV-leaf. **a** Typical internal structure of a real leaf. The vascular bundles uniformly distribute liquid water throughout the whole surface of the leaf. Effective transpiration cooling protects the photosynthetic process. **b** Internal structure of the bio-inspired transpiration structure. Hydrophilic fibre bundles and hydrogel cells are used to mimic the vascular bundles and sponge cells. **c** Exploded view of the transpiration structure. The biomimetic transpiration (BT) layer is constructed of bamboo fibre bundles and packed hydrogel cells. The root of the fibre bundles is soaked in bulk water. **d** Diagram and working principle of the PV-leaf transpiration structure. Water flows from the root to the hydrogel cells driven by capillary and osmotic processes. The water molecules in the molecular mesh then evaporate, removing PV heat. **e** Photograph of the single PV-leaf prototype.

As illustrated in Fig. 1a, a typical plant leaf structure comprises photosynthetic cells, vascular bundles (veins), sponge cells and stomata, cuticle and epidermis^28^. Within the plant, a flow of liquid water from the soil to the leaves is driven by capillary forces and osmotic pressure. Microchannels in the vascular bundles of a leaf are then responsible, and highly effective, in moving and distributing the water throughout the leaf^29^. The water then evaporates on the surfaces of cells during the transpiration process.

Inspired by the effective transpiration process and structure of natural leaves, we have designed a biomimetic transpiration structure for the PV-leaf as shown in Fig. 1b. In this design, a biomimetic transpiration (BT) layer is attached to the back of a solar PV cell in order to remove the heat generated in the cell. Natural bamboo fibre bundles are employed to mimic the vascular bundles in transporting and distributing liquid water over the cell’s surface, while hydrogel cells with large specific surface area and excellent water absorption performance^30,31^ are used to mimic the sponge cells in providing effective evaporation. Fig. 1c shows the configuration of the PV-leaf transpiration structure, which comprises a BT layer (~1 mm thick) and a supporting mesh (0.5 mm thick) connected to the underside of a PV cell layer (~150 μm thick) over an effective area of 10 × 10 cm^2^. In the BT layer, around 30 branches of bamboo fibre bundles are homogeneously embedded into the potassium polyacrylate (PAAK) superabsorbent polymer (SAP) hydrogel cells, distributing water over the entire area covered by the BT layer. The ends of the fibre branches are gathered together and soaked in water. The step-by-step fabrication method of the PV-leaf is shown in Supplementary Fig. 1. The detailed circuit diagram of the electrical measuring system used for the PV-leaf in this work is shown in Supplementary Fig. 2.

An overview of the main energy and mass transfer processes inside the PV-leaf are illustrated in Fig. 1d. Solar energy absorbed by the PV cell is converted into electricity and internal (thermal) energy. The heat then conducts away from the PV cell to the SAP hydrogel cells, which are tightly attached to the back of the PV cells in order to promote good thermal contact. The water molecules in the SAP hydrogel cells gain sufficient kinetic energy to overcome the intermolecular hydrogen bonds, enabling them to evaporate, thus removing thermal energy. Driven by capillarity and osmosis formed by the SAP polyelectrolyte hydrogel cells^32^, liquid water flows continuously from the water tank to the SAP hydrogel cells in order to supplement the water lost by evaporation. A proof-of-concept PV-leaf prototype, as shown in Fig. 1e, was constructed, with an effective PV area of 10 × 10 cm^2^, protected by covering a 0.7-mm-thick high-transmittance glass layer.

### PV-leaf transpiration performance

**Fig. 2:**
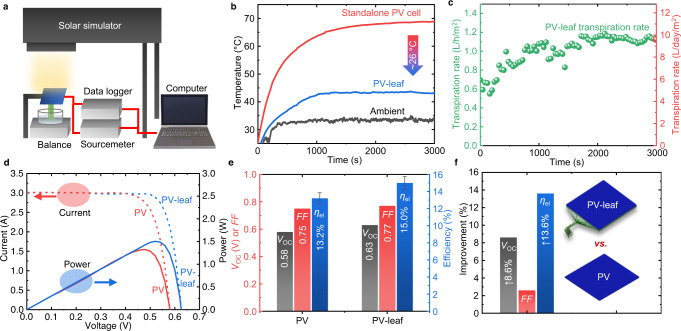
Transpiration performance of the PV-leaf. **a** Diagram of the testing platform. A calibrated solar simulator was used to generate sunlight with an average irradiance of *G* = 1000 W/m^2^. The detailed circuit diagram of the electrical measuring system used for the PV-leaf in this work is shown in Supplementary Fig. 2. **b** Temperature profile of the PV-leaf compared to that of a standalone conventional PV cell. A significant temperature reduction of ~26 °C was observed in the test. **c** Transpiration rate of the PV-leaf is around 1.1 L/h/m^2^ (left y-axis), equivalent to 9.7 L/day/m^2^ (right y-axis) according to a whole-day simulation in the Supplementary Information. **d** *V–I* and *P–I* profiles of the PV-leaf and standalone PV cell. Significant positive shifts in the curves were observed in PV-leaf which benefitted from the cooling effect. **e** Comparisons of crucial electrical parameters (open-circuit voltage *V*_oc_, fill factor *FF* and electrical efficiency *η*_el_) of the PV-leaf and conventional PV cell. **f** Relative electrical improvements of the PV-leaf compared to the conventional PV cell.

The transpiration performance of the PV-leaf was tested under a solar simulator with an irradiance of *G* = 1000 W/m^2^ (measured by a pyranometer), with no wind, and then compared to that of a standalone PV cell of the same material. The standalone PV cell was also covered and protected by a 0.7-mm-thick high-transmittance glass layer, but without any insulation or back sheet on the back of the cell (as shown in Supplementary Fig. 1). The standalone PV cell is cooled by natural air convection. The connection between the PV cell and a sourcemeter is shown in Supplementary Fig. 2. The configuration of the laboratory testing platform is shown in Fig. 2a and the calibration result of the solar simulator is presented in Supplementary Fig. 3. The ambient temperature and relative humidity in the laboratory were 33.5 °C and 10%, respectively. During the tests, the operating temperature, open-circuit voltage (*V*_oc_), fill factor (*FF*) and electrical efficiency (*η*_el_) of the PV cells in the PV-leaf and standalone PV cell were measured.

The standalone PV cell reached a temperature of 68.8 °C whereas the PV-leaf with biomimetic transpiration cooling reached a temperature of only 43.2 °C, as shown in Fig. 2b, such that the operating temperature was significantly reduced (by ~26 °C). Assisted by a continuous supplement of liquid water driven by capillarity, the biomimetic transpiration process was stable and the PV-leaf temperature reached a steady value after about *t* = 1000 s. The transpiration rate firstly increased and then stabilised at around 1.1 L/h/m^2^, as shown in Fig. 2c, where the transpiration rate is defined in this work as the (volumetric) evaporation rate (or flow rate of delivered liquid water) per unit collection area.

Of note is that the transpiration rate increased significantly as the PV-leaf operating temperature increased (Supplementary Fig. 4). This interaction between the operating temperature and transpiration rate allowed for effective control of the PV-leaf temperature and contributed to the stabilisation of this temperature after a short transient. It is of interest to recall that the experiments in this study were conducted in a no-wind laboratory environment, and that the PV-leaf temperature will be expected to further reduce due to convection and the effect of wind speed in an outdoor environment. To gain further insight into the role of different experimental variables (i.e. wind speed, relative humidity, water tank temperature, and thickness of the BT layer), a 3-D model was developed and validated against the experimental results (Supplementary Fig. 5). Simulation results in Supplementary Fig. 6 show that the PV-leaf temperature can be lower than the ambient temperature when the wind speed is higher than 1.5 m/s. Supplementary Fig. 6 also shows the effects of the relative humidity and of the water tank temperature on the cooling performance of the PV-leaf. The temperature reduction (i.e. temperature difference between the PV-leaf and the standalone PV cell) decreases almost linearly from ~26 °C to 0 °C as the relative humidity increases from 10% to 100%. The temperature of the non-insulated water tank is close to the ambient temperature and has a slight influence on the cooling performance.

Benefiting from the lower PV cell operating temperature relative to a standalone PV cell at otherwise similar conditions, the electrical performance curves of the PV-leaf can be observed in Fig. 2d to shift towards higher values, specifically, with the open-circuit voltage increasing from 0.58 V to 0.63 V, the fill factor increasing from 0.75 to 0.77, and the electrical efficiency increasing from 13.2% to 15.0%, as shown directly in Fig. 2e. The open-circuit voltage and electrical efficiency of the PV-leaf were improved by 8.6% and 13.6% relative to the standalone PV cell as shown in Fig. 2f, acting to demonstrate the significant advantages of the PV-leaf concept.

**Fig. 3:**
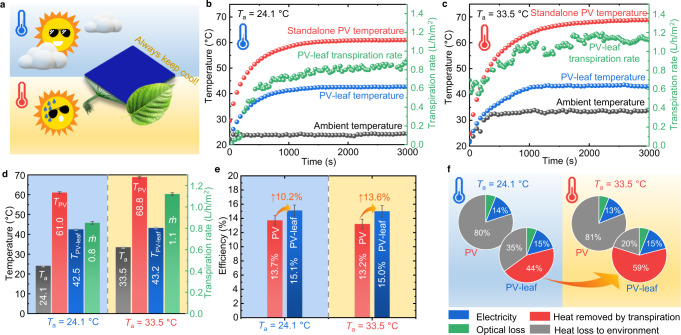
Passive control of the PV-leaf transpiration operation and performance at different ambient temperatures. **a** ‘Always keep cool’. The PV-leaf has the capability of passive temperature control, working as a real leaf. **b** Temperature profile and transpiration rate of the PV-leaf at a normal ambient temperature of 24.1 °C. **c** Temperature profile and transpiration rate of the PV-leaf at a higher ambient temperature of 33.5 °C. **d** Temperatures (*T*_PV_ and *T*_PV-leaf_) and transpiration rates (*ṁ*) of the standalone PV cell and PV-leaf at different ambient temperatures (*T*_a_). PV-leaf temperature increased by less than 1 °C as the ambient temperature increased by ~9 °C, benefiting from the passively controlled transpiration rate. **e** Comparison of electrical efficiencies of the standalone PV cell and PV-leaf. The advantage of the PV-leaf over the standalone PV is more significant at a higher ambient temperature. **f** Energy allocation analyses at different ambient temperatures. Transpiration is able to remove a large amount of heat (0.59*G*) in the PV-leaf at the ambient temperature of 33.5 °C, which accounts for ~75% of the total heat.

The transpiration rate of a natural leaf is passively controlled to keep the leaf temperature stable over a range of ambient conditions with minimum water consumption^27^. The PV-leaf was tested at two different ambient temperatures of 24.1 °C and 33.5 °C, so as to investigate the passive control effect of the PV-leaf. The performance of the PV-leaf alongside a reference standalone PV cell at the lower ambient temperature (24.1 °C) is shown in Fig. 3b, where it can be seen that, at these conditions, the standalone PV cell reached 61.0 °C while the PV-leaf was only at 42.5 °C with a transpiration rate of 0.8 L/h/m^2^. When the ambient temperature was increased from 24.1 °C to 33.5 °C, the standalone PV cell temperature increased from 61.0 °C to 68.8 °C, as shown in Fig. 3c, i.e. the standalone PV cell temperature increased by 7.8 °C as the ambient temperature increased by 9.4 °C. Interestingly, the PV-leaf temperature increased by a slightly smaller margin, from 42.5 °C to 43.2 °C (by less than only 1 °C), as the ambient temperature increased from 24.1 °C to 33.5 °C. The transpiration rate of the PV-leaf increased by 37% (from 0.8 to 1.1 L/h/m^2^) as the ambient temperature increased, providing additional cooling power in the hotter environment. Thus, the PV-leaf is able to keep cool over a range of ambient temperatures. The temperatures and transpiration rates of the PV-leaf at the two different ambient temperatures are summarised in Fig. 3d and compared to those of the standalone PV cell.

As shown in Fig. 3e, the electrical efficiencies of the standalone PV cell and PV-leaf were 13.7% and 15.1% when the ambient temperature was 24.1 °C, i.e. the electrical efficiency was improved by 10.2% (in relative terms) as a result of the applied biomimetic transpiration cooling. The electrical efficiency of the standalone PV cell decreased from 13.7% to 13.2% as the ambient temperature increased from 24.1 °C to 33.5 °C, while only a slight efficiency degradation of 0.1% was observed for the PV-leaf. The electrical efficiency of the PV-leaf was 13.6% (relative) higher than that of the standalone PV cell when the ambient temperature was 33.5 °C. The improvement in the electrical efficiency of the PV-leaf relative to the standalone PV cell is more significant at the higher ambient temperature.

Energy allocations in the standalone PV cell and in the PV-leaf at different ambient temperatures are shown in Fig. 3f. When the ambient temperature was 24.1 °C, all the heat generated in the standalone PV cell, accounting for 80% of the incident solar energy (i.e. 0.80*G*), was dissipated to the environment by weak natural convection and radiation cooling. Similar heat (0.81*G*) was generated in the standalone PV cell when the ambient temperature was 33.5 °C. The standalone PV cell thus raised to a higher operating temperature when the ambient temperature increased from 24.1 °C to 33.5 °C in order to dissipate this amount of heat to the hotter environment. PV-leaf transpiration removed a significant amount of heat (0.44*G*) when the ambient temperature was 24.1 °C, accounting for 56% of the total PV heat. Interestingly, the heat removed by the PV-leaf transpiration increased significantly from 0.44*G* to 0.59*G* when the ambient temperature increased from 24.1 °C to 33.5 °C. The cooling power reached 590 W/m^2^, thus removing 75% of the total heat when the ambient temperature was 33.5 °C. The PV-leaf kept cool with a slight temperature increase of <1 °C when the ambient temperature increased by 9.4 °C. The PV-leaf has shown its ability to passively control its transpiration rate, thus keeping its operating temperature at a low level to ensure a satisfying electrical efficiency. A series of outdoor experiments were conducted on a sunny summer day in London (shown in Supplementary Fig. 7). These outdoor test results also demonstrated that the PV-leaf could passively control its transpiration rate in a real outdoor environment and had a stable diurnal (whole day) cooling performance.

**Fig. 4:**
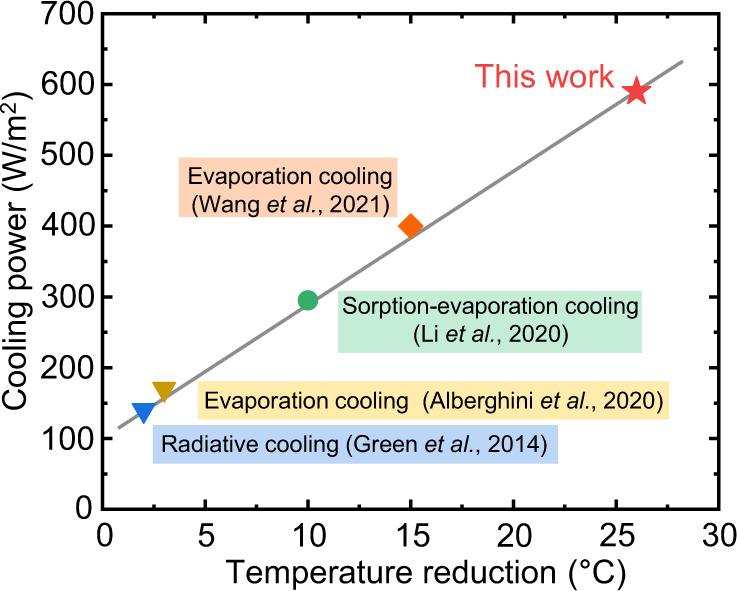
Comparison of the PV-leaf transpiration cooling performance of this study with other emerging PV thermal management technologies. The cooling power of the biomimetic transpiration in this work is significantly higher than that of radiative cooling (Green et al.^21^), atmospheric-water sorption-evaporation cooling (Li et al.^24^), and evaporation cooling (Alberghini et al.^33^ and Wang et al.^34^.).

The biomimetic transpiration process in the PV leaf can remove 590 W/m^2^ from the PV cell, which is significantly higher than that achieved by cutting-edge radiative cooling methods^21^ (40–140 W/m^2^), atmospheric-water sorption-evaporation cooling^24^ (295 W/m^2^) and other emerging evaporation cooling solutions^33,34^ (180–400 W/m^2^), as shown in Fig. 4. Interestingly, the temperature reduction increases linearly as the cooling power increases. The operating point of the PV-leaf (the pentagram in Fig. 4) falls on the projected line that also passes through the previous studies in the same figure, indicating but also confirming the potential of the PV-leaf concept. In more detail, and with specific reference to other solar cell evaporation cooling designs reported previously in literature: (i) the biomimetic transpiration structures of stacked hydrogel cells proposed in our study can provide more effective water transport owing to the hierarchical porous structure, while current solar cell evaporative cooling designs in the literature are based on homogeneous porous structures; and (ii) the stacked PAAK hydrogel cells can provide more effective water evaporation owing to their 3-D evaporation structure (i.e. water evaporation takes place over 3-D stacked cell surfaces), while current solar cell evaporative cooling designs are based on 2-D evaporation structures (i.e. with evaporation over flat surfaces).

### Compatibility of utilising saline as the coolant

The PV-leaf has proven to be effective at removing the heat generated in the PV cell via biomimetic transpiration and to be able to control its transpiration rate to minimise water consumption, however, it could be challenging to provide sufficient freshwater as coolant for areas where freshwater resources are still scarce. Therefore, it is essential to investigate the performance of the PV-leaf using alternative coolants for transpiration, especially seawater (after pre-treatment such as alumina activation), which is abundant and account for 97% of the Earth’s water. This abundance can provide enough low-cost, sustainable coolant for a substantial capacity of PV-leaf plants.

**Fig. 5:**
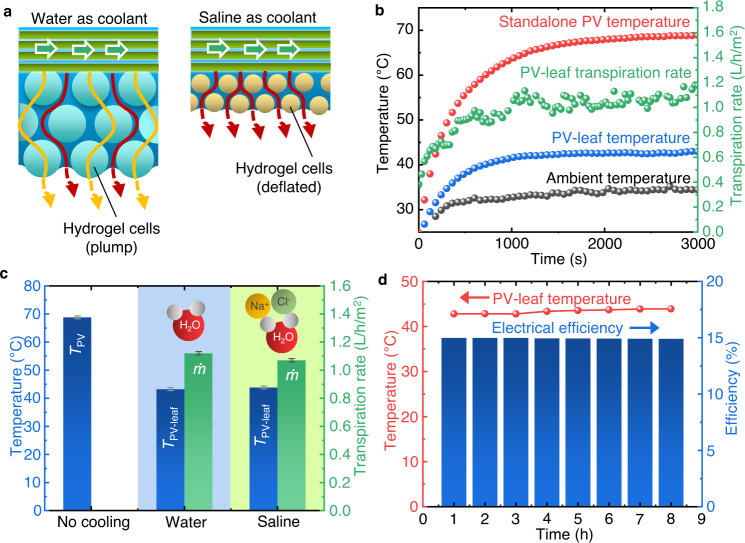
Performance of the PV-leaf using saline (3.5%wt) as the working fluid. **a** Water transportation pathways for water or saline as the coolant. Red lines: water transportation through micro-gaps between the cells; yellow lines: water transportation through the hydrogel polymer framework. **b** Temperature profile and transpiration rate of the PV-leaf using the saline as working fluid. **c** PV-leaf temperature (*T*_PV-leaf_), and transpiration rate (*ṁ*) for different working fluids of water and saline. PV-leaf has shown the compatibility of utilising saline as the working fluid. **d** Performance of PV-leaf using saline as the working fluid under an 8-h test with peak solar irradiances (*G* = 1000 W/m^2^).

The PAAK hydrogel cells are plump when using water as the coolant. Of note is that water can flow through the porous BT layer via either the micro-gaps between the hydrogel cells (the red lines in Fig. 5a) or through the hydrogel polymer framework (the yellow lines in Fig. 5a). PAAK hydrogel cells shrink when saline acts as a coolant, due to the infiltration of cations into the polymer framework, and the resulting reduction in the anion–anion electrostatic repulsion and osmotic pressure difference^35^. Although the water conductivity inside the deflated hydrogel cells is limited when using saline as a coolant, water can still flow efficiently through the micro-gaps between the hydrogel cells, as shown on the right in Fig. 5a (the red lines). The high efficiency of water transport in the hierarchical porous hydrogel was also investigated and validated by ref. ^31^.

Figure 5b shows the temperature profile and transpiration rate of the PV-leaf when using saline solution as the working fluid. In this study, we used a saline solution with a NaCl concentration of 3.5%wt to simulate seawater. Interestingly, both the cooling performance and transpiration rate of the saline was similar to those of the freshwater. The PV-leaf using saline coolant was effectively and stably cooled to 43.8 °C, only slightly higher than the 43.2 °C when using water as the coolant, which gives confidence that the PV-leaf has compatibility in using saline as a coolant without significantly compromising its transpiration cooling performance. Both the freshwater and saline were able to reduce the operating temperature of the PV cell from ~69 °C without transpiration to ~43 °C with transpiration, as shown in Fig. 5c. The results prove that porous BT structures made of stacked PAAK hydrogel cells can efficiently transport water to ensure efficient transpiration cooling when saline is used as a coolant.

We examined the performance of the PV-leaf over 8 h of continuous operation using saline under a peak solar irradiance (*G* = 1000 W/m^2^) in an effort to simulate its operation and performance during a very sunny day. The 8-h long test results demonstrated that the PV-leaf was able to deliver a stable, constant rate of cooling that maintained the PV-leaf at ~43 °C, indicating that the amount of micro salt crystals possibly formed in the porous BT layer did not hinder the effective transport of water during these longer-term tests. No obvious salt crystallisation was observed on the outer surface of the mesh after 8 h of continuous testing (as shown in Supplementary Fig. 8). The space of the micro-gaps in the porous BT layer was enough to store the micro salt crystals generated during the 8-h test.

The BT layer is then allowed to reject salt ions back to the bulk saline ‘overnight’ by diffusion^36^. Of note is that the efficiency of the natural salt rejection may not be high enough to reject all salts back to the tank ‘overnight’ due to the aspect ratio of the water transportation channels (i.e. the bamboo fibres)^37^. Therefore, low-cost regular maintenance, such as additional low-cost seawater flushing^38^, can be used to remove the accumulated salt from the BT layer, as shown in Supplementary Fig. 9. The BT layer can be refreshed by quick and low-cost flushing, and then be ready for operation the next day.

The ability to utilise saline as a coolant enables the PV-leaf to be cost-effective and feasible even in areas facing a shortage of freshwater. The 8-h tests provide some evidence of the feasibility of utilising saline as a transpiration coolant. Further research should focus on optimising the salt rejection structure (e.g. by reducing the characteristic length scale of the evaporator^37^ or employing a zero-liquid-discharge design^39^) to ensure stability in long-term operation. We selected PAAK hydrogel cells to construct to the porous BT layer, as PAAK is an eco-friendly, low-cost and commercially-available material. Although PAAK hydrogel cells shrink in saline, the porous BT layer is still able provide effective transpiration cooling. Polyelectrolyte hydrogel with better salt rejection performance^32^ can be considered in future work, but the cost and impact on the environment also need to be justified. Seawater pre-treatment (such as physical pre-treatment by using activated alumina) is also important prior to the evaporation or desalination process, to relieve corrosion and fouling during long-term operation. The effect of scaling formed by magnesium species in real seawater on PV-leaf performance also requires additional attention^39^.

### Synergistic generation of electricity, heat and clean water

Meeting the growing energy demand sustainably remains a global challenge. In addition to electricity, heat is another important energy demand^40^. Freshwater scarcity is also a key challenge for global sustainable development^41–43^. Nearly two-thirds of the world’s population is experiencing severe water scarcity during at least part of a year^44^. It is crucial to meet the combined growing demands of electricity, heating and freshwater sustainably and efficiently.

**Fig. 6:**
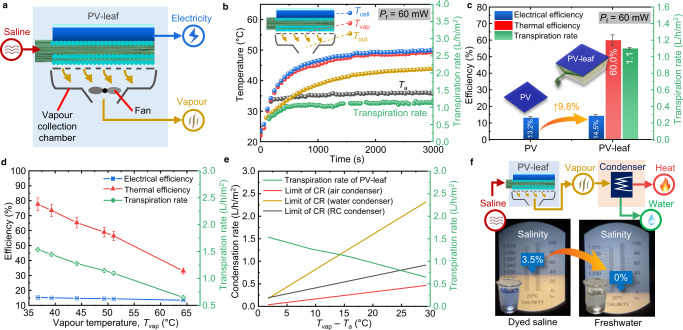
Synergistic generation of electricity, heat and clean vapour of the hybrid multi-generation PV-leaf. **a** Schematic of the PV-leaf. A chamber was attached below the BT layer to collect clean vapour. **b** Temperature profile (temperatures of the solar cell *T*_cell_, the vapour on the interface *T*_vap_, and the outlet of the chamber *T*_out_) and transpiration rate of the PV-leaf when the ventilation fan power is *P*_f_ = 60 mW. **c** Comparison of outputs of the PV-leaf and the standalone PV cell when *P*_f_ = 60 mW. **d** Electrical efficiency, thermal efficiency and transpiration rate of the PV-leaf as a function of the vapour temperature *T*_vap_, which was adjusted by changing the ventilation fan power. The vapour temperature *T*_vap_ increases as the ventilation power decrease. **e** Different condensation technologies and corresponding condensation rate (CR) limits. A separate condenser is needed to condense the vapour. The limits of the air-based condenser (*h*_c_ = 10 W/m^2^/K) and radiative cooling (RC) condenser are provided by ref. ^49^. The limit of the water-based condenser (*h*_c_ = 50 W/m^2^/K) is assumed to be five times that of the air-based condenser. **f** The salinity of the input saline and output freshwater tested by a refractometer. A separate flat-plate water-based condenser was used to condense the vapour generated by the PV-leaf, as shown in Supplementary Fig. 10. The saline was dyed to blue to help visualise the cleaning effect.

Forests are highly important in regulating the world’s temperatures and producing freshwater flows^45^. Over 40% of rainfall over land comes from evapotranspiration, i.e. evaporation from soil and transpiration from trees. Rainforest transpiration is even crucial for initiating the local transition of dry-to-wet season^46^. Transpiration plays important roles in not only solar photosynthesis conversion but also in freshwater generation in nature, which strongly inspire us to explore the potential of synergistic energy-water cogeneration of the PV-leaf. The PV-leaf transpiration structure has proven to be effective at utilising saline for transpiration, generating electricity at high efficiency. Of note is that transpiration generates clean vapour, which can be further converted into freshwater and thermal energy through condensation. A vapour collection chamber is attached below the BT layer to collect the clean vapour, as shown in Fig. 6a. The chamber was packed with a thermal insulation layer to reduce the heat loss to the environment in the test. A small ventilation fan was used to draw out the vapour from the chamber. The power of the ventilation fan can be adjusted to control the performance of the PV-leaf. Of note is that the proof-of-concept PV-leaf can only generate electricity and clean water vapour (without having an in-built condensation function). This section focuses on investigating the electrical, thermal and vapour-generation performance of the PV-leaf. An additional downstream condenser is needed to condense the vapour further to produce fresh water and release thermal energy.

Figure 6b shows the temperature profile and transpiration rate of the PV-leaf under solar irradiance of 1000 W/m^2^ with 60 mW of power consumed by a ventilation fan. The temperatures of the solar cell *T*_cell_, the vapour on the interface *T*_vap_, and the outlet of the chamber *T*_out_ were 49.9, 48.5, and 44.0 °C, respectively, while the transpiration rate (i.e. clean vapour generation rate) was around 1.1 L/h/m^2^. The outlet temperature *T*_out_ was ~4.5 °C lower than the vapour temperature *T*_vap_ since the unavoidable heat loss of the chamber. The outputs of the standalone PV cell and the PV-leaf are summarised and compared in Fig. 6c. The PV-leaf produced additional heat (in terms of vapour) with a thermal efficiency of 60%, while its electrical efficiency (14.5%) remained higher than the standalone PV cell (13.2%). The thermal efficiency here is defined as^47,48^:

1 $${\eta }_{{{{{{\rm{th}}}}}}}=\frac{\dot{m}{c}_{{{{{{\rm{P}}}}}}}\left({T}_{{{{{{\rm{vap}}}}}}}-{T}_{{{{{{\rm{t}}}}}}}\right)+\dot{m}{\lambda }_{{{{{{\rm{vap}}}}}}}}{G\cdot A}$$

where $\dot{m}$ is the transpiration rate, *c*_p_ the specific heat capacity of water, *T*_t_ the bulk water temperature in the tank, *A* is the effective area of the PV-leaf. The water evaporation enthalpy of the hydrogel, *λ*_vap_, was measured by the method outlined in ref. ^30^. Detailed experimental procedure and results are provided in the supplementary file (Supplementary Fig. 12).

The overall solar utilisation efficiency (electrical + thermal) of the PV-leaf reached 74.5%, which was significantly higher than the standalone PV cell (only 13.2%), due to the fact that the latter has no output other than the generated electricity. In addition to the electrical and thermal outputs, around 1.1 L/h/m^2^ of saline was evaporated to clean vapour in the PV-leaf. Although the ventilation fan consumed 60 mW of electricity, this accounted for only ~4% of the electricity generated by the PV-leaf.

The condensation rate of the vapour depends on not only the heat transfer efficiency of the condenser, but also the vapour temperature^49^. The temperature of the vapour generated by the PV-leaf can be flexibly adjusted by changing the ventilation fan power. As the ventilation power decreases, the discharge rate of the vapour from the chamber decreases, resulting in a decrease in the transpiration rate and thus an increase in the vapour temperature, and vice versa. The vapour temperature *T*_vap_ could be adjusted over a large range from ~36 °C to ~64 °C, as shown in Fig. 6d. Both the electrical and thermal efficiencies, and the transpiration rate of the PV-leaf decreased nearly linearly as the operating temperature *T*_vap_ increased in the experiments. Users can flexibly select their desired working conditions depending on their specific demands for electricity, thermal energy, but also freshwater. The low-grade thermal energy can be used for domestic space heating or preheating of domestic hot water.

Of note is that low-temperature vapour condensation remains a challenge^49^. In order to estimate the water productivity of the PV-leaf, we summarise the limits of condensation rates (CR) of different condensation technologies in Fig. 6e (i.e. air-, water- and radiative-cooling-based condensers). Zhou et al.^49^. proposed a radiative cooling condenser which has a better performance than the conventional air-based condenser. Water-based condenser has a higher heat transfer coefficient (*h*_c_), and thereby a higher CR limit. Although a larger amount of vapour can be generated when the vapour temperature is close to the ambient temperature, the low-temperature vapour is challenging to be condensed with current condensation technologies. The vapour with a higher temperature can be more easily condensed, but the transpiration rate of the PV-leaf significantly decreases as the vapour temperature increases. The cross point between the CR curves and the transpiration rate curve in Fig. 6e indicates the maximum CR (i.e. water productivity) of the PV-leaf, i.e. around 1.1 L/h/m^2^ by using the water-based condenser.

Even though condensation of low-temperature vapour is out of the main research scope of this study, we fabricated a tailored flat-plate water-based condenser to condense the vapour generated by the PV-leaf, as shown in Supplementary Fig. 10, and to check the desalination effect. The salinity effectively decreases from ~3.5% (the saline) to ~0% (the condensed water), as shown in Fig. 6f. The saline was also dyed to observe the cleaning process visibly.

The conventional PV-thermal desalination system also can cogenerate electricity, thermal energy and clean water^50^, which typically consists of a hybrid PV-thermal solar collector and a separate desalination module. Comparing to the conventional PV-thermal desalination system, the desalination process of the PV-leaf occurs below the PV cells without the need of separate desalination modules. The passive control and self-pumping capability of the PV-leaf enables it to automatically adjust the water mass flow rate, thus eliminating the need for additional pumps and control units.

## Discussion

We have presented a bio-inspired PV-leaf design that has the potential to address the critical need for the effective thermal management of PV panels, while delivering additional useful outputs of both thermal energy and freshwater. The PV-leaf is free from complicated heat exchangers, membranes, pumps and control systems. Demonstration results showed that the bio-inspired transpiration structure implemented within the PV-leaf can remove ~590 W/m^2^ heat from the PV cell under a solar irradiance of 1000 W/m^2^, decreasing the PV cell temperature by ~26 °C and leading to a significant improvement of its electricity output by ~14%. Compared to previous studies on transpiration cooling^51^, the solution in this work does not require pump, control unit, expensive porous materials, and is able to cool the target surface to a significantly lower temperature, which is suitable for multi-generation applications, as well as applications of thermal management for PV cells. Both indoor and outdoor testing demonstrated that a PV-leaf can passively control its transpiration rate to adapt to different ambient temperatures and solar irradiance. The PV-leaf has shown a strong compatibility for utilising seawater, which is far more abundant than freshwater as its working fluid without significantly compromising its thermal management performance. Similar to tree transpiration, the PV-leaf is able to control the transpiration rate passively in response to changes to its internal conditions and the ambient temperature. Simulation results show that PV-leaf has better transpiration performance in hot and dry climates.

The PV-leaf can also synergistically produce an additional 1.1 L/h/m^2^ of freshwater under a solar irradiance of 1000 W/m^2^, while improving the electrical efficiency of the device by ~10% and generating considerable additional useful thermal energy from the heat recovered from the solar cell. Assuming a PV electrical efficiency of 20% and 100 equivalent sunny days in a year, the projected 8.5 TW of installed PV panels in 2050 would produce over 40 billion m^3^ of freshwater each year if the panels were to employ a PV-leaf structure, significantly relieving the stress of global water scarcity.

We employed widely-available, low-cost and environmentally-friendly materials and components to fabricate the key components of the PV-leaf, avoiding complex and costly customisation, which assists in ensuring that the additional electricity generation has a value greater than, and that more than compensates for, the cost of additional materials and components. Specifically, the capital cost of the additional transpiration components (hydrogel, fibre bundle, supporting mesh and piping) of the PV-leaf relative to a conventional standalone PV panel is estimated to be ~1.1 $/m^2^, based on available bulk prices (Supplementary Table 1), which is only ~2% of the price of a commercial PV panel (~55 $/m^2^). The payback time of the additional components is thus less than half a year. Given current predictions for the global PV capacity to reach over 22 TW by 2050, and assuming that 30% of the PV panels have access to water resources as coolant, PV-leaf designs promise to generate an additional ~650 GW of power globally, which is close to the current global PV installed capacity.

As a future perspective beyond the main scope of this work, the PV-leaf concept can be upscaled to larger-scale collectors, beyond which even larger solar plants of a commercial size can be divided into several small areas allocated to separate, interconnected PV-leaves (e.g. see Supplementary Fig. 11). We hope that this work can help motivate further studies on commercially relevant PV panel cooling designs, as well as other systems, which may benefit from biomimetic transpiration cooling.

Overall, the high-efficiency and low-cost bio-inspired hybrid PV-leaf shows great promise for significantly increasing the capacity of solar installations, without relying on complicated and expensive heat transfer elements and other components, enabling the potential of simultaneously solving the global challenges of increasing energy demands and freshwater scarcity, and accelerating the race to net-zero.

## Methods

### PV-leaf fabrication

The BT layer was constructed of PAAK hydrogel cells and bamboo fibre bundles. Commercial PAAK particles were purchased from Wastland. First, 1.0 g PAAK particles were dispersed in 200.0 g of distilled water. The mixture was stirred for 5 min and allowed to stand for 2 h at normal laboratory conditions. The fully swelled hydrogel cells had diameters in the range ~2–5 mm. A 5-mL syringe was used to mill the hydrogel cells from a large size (~2–5 mm) to a smaller size (~0.2 mm). The water evaporation enthalpy of the hydrogel cells was measured by the method described in ref. ^30^. A stainless-steel woven wire mesh was used to support the BT layer. In total, around 30 branches of bamboo fibre bundles with diameters of ~1 mm were fixed uniformly over the surface of the stainless-steel woven wire mesh. The distribution of the fibre branches is shown in Fig. 1c. The hydrogel cells were then uniformly pasted on the stainless-steel woven wire mesh and wrapped around the fibre bundles. The BT layer had a thickness of ~1 mm and an effective area of 10 × 10 cm^2^, and was attached to the back of the 150-μm-thick monocrystalline silicon solar cell (SunPower). The back surface of the solar cell was coated with ultra-thin (<50 μm thick) electrical insulation and an anti-corrosion coating (3M Scotch 1601) to avoid any impact of corrosion or short circuiting on the PV efficiency. A 0.7-mm-thick high transmittance glass (Schott Borofloat) with 94% transmittance was used to cover the solar cell for protection, and 2-mm-thick aluminium frames were used to clamp the layers together. The effective solar absorption area of the PV-leaf was 10 × 10 cm^2^. The vapour collection chamber (10.5 × 10.5 × 1.5 cm^3^) was made of 3-mm-thick polycarbonate plates. The power of the DC brushless ventilation fan was controlled by a DC power supply.

### PV-leaf indoor test

The root of the fibre brunches was soaked in the covered cup filled with water or saline (3.5%wt) during the tests. The prototypes were tested under 1000 W/m^2^ of simulated sunlight in a solar simulator (K. H. Steuernagel Lichttechnik) calibrated by a first-class pyranometer (KIPP & ZONEN-CMP6 with an accuracy of ±0.5%). In order to calculate the PV electrical efficiency, a high-precision source metre (Keithley-2460 with an accuracy of ±0.02%) with *I*–*V* tracing software (TestScriptBuilder) was used to measure *I*–*V* curves. A four-wire connection method was made to eliminate the effects of lead resistance when measuring the electrical performance. The mass loss of the water cup was measured by using a precision balance with a 1-mg resolution (Kern-PNS-600 with an accuracy of 0.001 g). The transpiration rate (i.e. rate of water mass loss) was then calculated using an in-house MATLAB code. Four calibrated T-type thermocouples (T.C. Direct with an accuracy of ±0.5 K) were attached uniformly over the back of the solar cell to measure the solar cell’s temperature. Two calibrated T-type thermocouples and a humidity meter (Habor with an accuracy of ±7%) were installed around the solar collector to measure the ambient temperature and relative humidity around the solar simulator area. The salinity of the saline solution was tested by a refractometer (ATC with an accuracy of ±0.1%). The experiments were conducted in a dedicated indoor solar-testing laboratory fitted with an air-conditioning system, which was operated using a set-point to control both the temperature and humidity at a suitable level for experiments and material storage. We also constructed a thermal insulation box around the solar simulator, which we used to increase the surrounding temperature to over 30 °C when simulating a hot climate.

### PV-leaf outdoor test

The outdoor testing platform is located on the roof of the Roderic Hill Building in the South Kensington Campus of Imperial College London (51° 29′ 58.11′′ N 0° 10′ 41.7648′′ W). Both the PV-leaf and standalone PV cell used for benchmarking/comparison purposes were oriented southwards and titled 30°. The methods used for measuring the operating temperature, mass flow rate and humidity were the same as the indoor tests. The solar irradiance was measured by a pyranometer (KIPP & ZONEN-CMP6). The wind speed was measured by a weather station (YOUSHIKO YC9388 with an accuracy of ±10%). The electronic balance was protected by a wind and sun shield.

### Simulation method

Comprehensive steady-state 3-D finite element models of the standalone PV cell and PV-leaf were developed using the COMSOL Multiphysics software (Version 5.6). All the geometric parameters and thermophysical properties are the same as the prototype. The evaporation curve of the PV-leaf (Supplementary Fig. 4) was applied to the model. The models have been validated against the experiment data and then used to investigate the daily performance of the standalone PV cell and PV-leaf (Supplementary Figs. 5 and 7).

### Reporting summary

Further information on research design is available in the Nature Portfolio Reporting Summary linked to this article.

## Supplementary information

Supplementary Information

Solar Cells Reporting Summary

## Data Availability

The data generated in this study are provided in the Supplementary Information and Source Data file, or from the corresponding author upon request. Source data are provided in this paper.

## Source data

Source Data

## Code availability

No custom code or mathematical algorithm in this study.
