# Supplementary material for: High-efficiency bio-inspired hybrid multi-generation photovoltaic leaf
Source: Nat Commun. 2023 Jun 8;14:3344. doi: 10.1038/s41467-023-38984-7 (PMC10250451; doi:10.1038/s41467-023-38984-7)
Supplement: Supplementary file 1 — Supplementary Information [file 41467_2023_38984_MOESM1_ESM.pdf]

High-efficiency bio-inspired hybrid multi-generation photovoltaic leaf

Gan Huang [1, §, *], *Jingyuan Xu* [1, §], *Christos N. Markides*[1,*]

[1] Clean Energy Processes (CEP) Laboratory, Department of Chemical Engineering, Imperial College London, U.K.

[§] These authors contributed equally.

*Corresponding authors: g.huang@imperial.ac.uk; c.markides@imperial.ac.uk

Supplementary figures

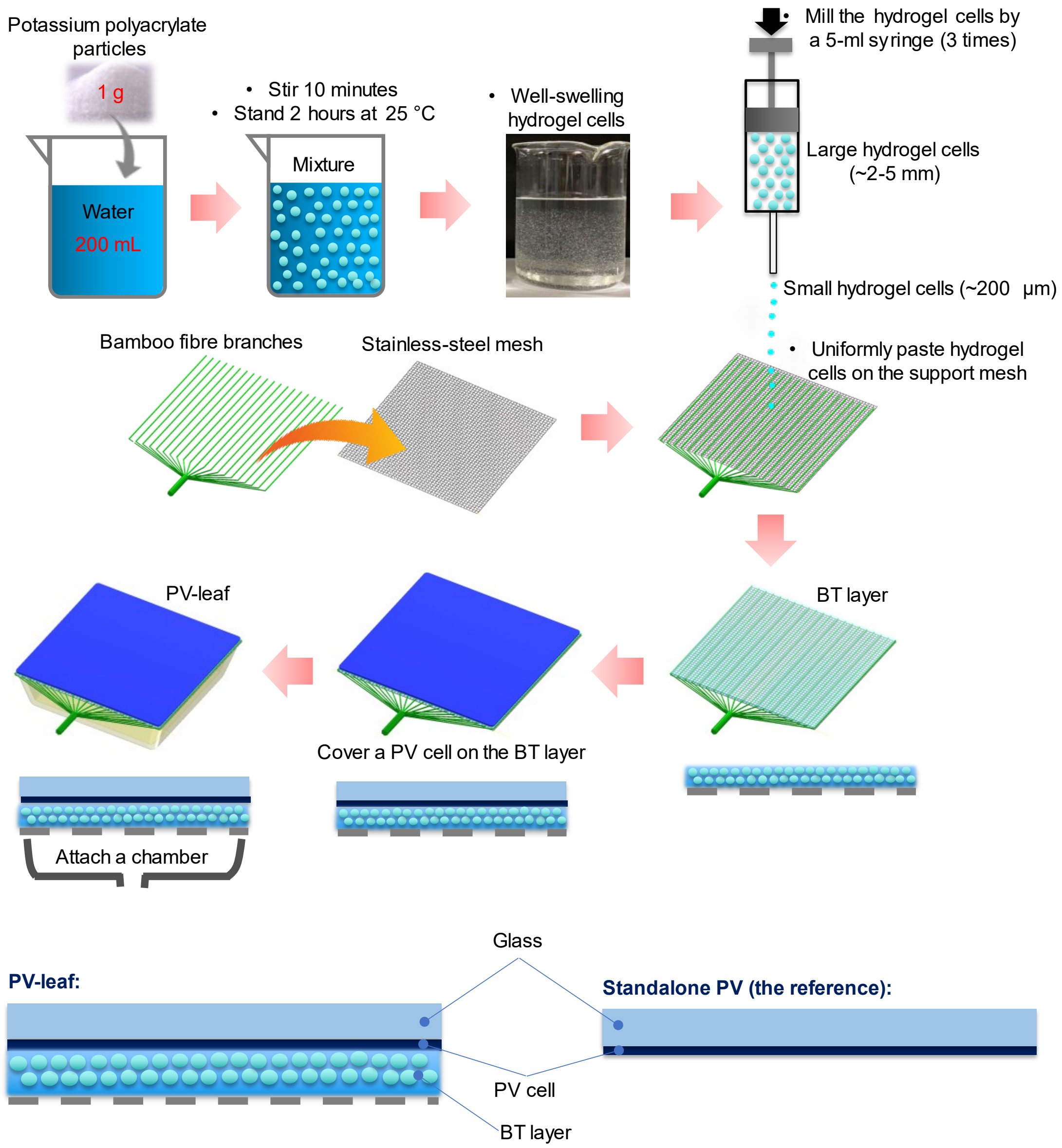

Supplementary Figure 1. Fabrication process of the PV-leaf. 1.0 g potassium polyacrylate particles were dispersed in 200.0 g of distilled water. The mixture was stirred for 5 min and allowed to stand for 2 h at normal laboratory conditions. The fully swelled potassium polyacrylate hydrogel cells had average diameters of ~2-5 mm. A 5-mL syringe was used to mill the hydrogel cells from a large size (~2-5 mm) to a smaller size (~0.2 mm). Bamboo fibre bundles with diameters of ~1 mm

were uniformly fixed onto the surface of a stainless-steel woven wire mesh. The small hydrogel cells were then pasted on the mesh and wrapped around the fibre bundles. The biomimetic transpiration (BT) layer, with a thickness of ~1 mm and an effective area of 10 × 10 cm^2, was attached to the back of a 150-μm-thick monocrystalline silicon PV cell. A vapour collection chamber (10.5 × 10.5 × 1.5 cm^3) with a ventilation hole is attached below the BT layer. A 0.7-mm-thick glass with 94% transmittance was used as a cover on the PV cell to protect it.

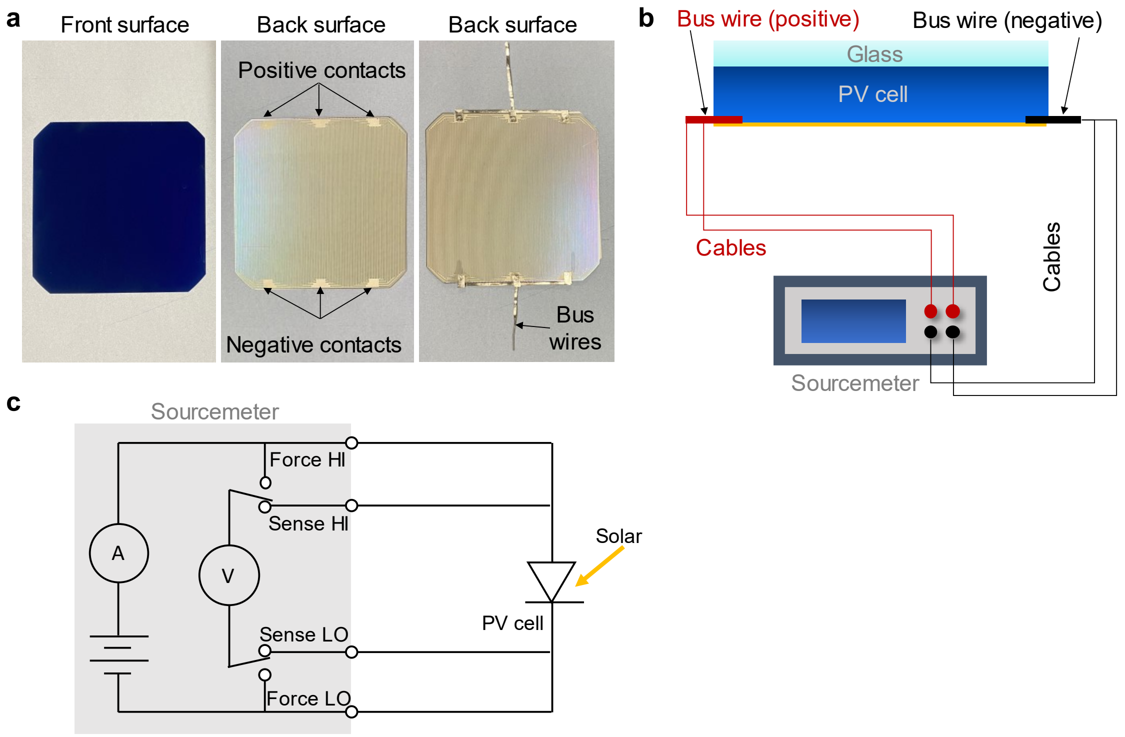

Supplementary Figure 2. Structure of the PV panel and connection to the sourcemeter. a, Photograph of the PV cell. Both the positive and negative contacts are on the back surface of the SunPower C60 solar cell. Bus wires were welded to the positive and negative contacts. **b**, Connection between the PV cell and sourcemeter. The bus wires were connected to the sourcemeter, which was controlled by a maximum power point tracking algorithm, by using the 4-wire method. The solar cell was then encapsulated with a 0.7-mm-thick glass (front surface), as well electrical insulation and anti-corrosion coating of approximate thickness 50-µm-thick (back surface). **c**, Circuit diagram of the electrical measuring system. A four-wire connection is made to eliminate the effects of the lead resistance. When connecting the leads to the solar cell, the Force LO and Sense LO connections were made to the cathode terminal. The Force HI and Sense HI connections were made to the anode. A maximum power point tracking algorithm was used to identify the electrical operating point, including the voltage, current, power output and efficiency. Detailed guidance on the four-wire connection method can be found the technical report by Keithley[1].

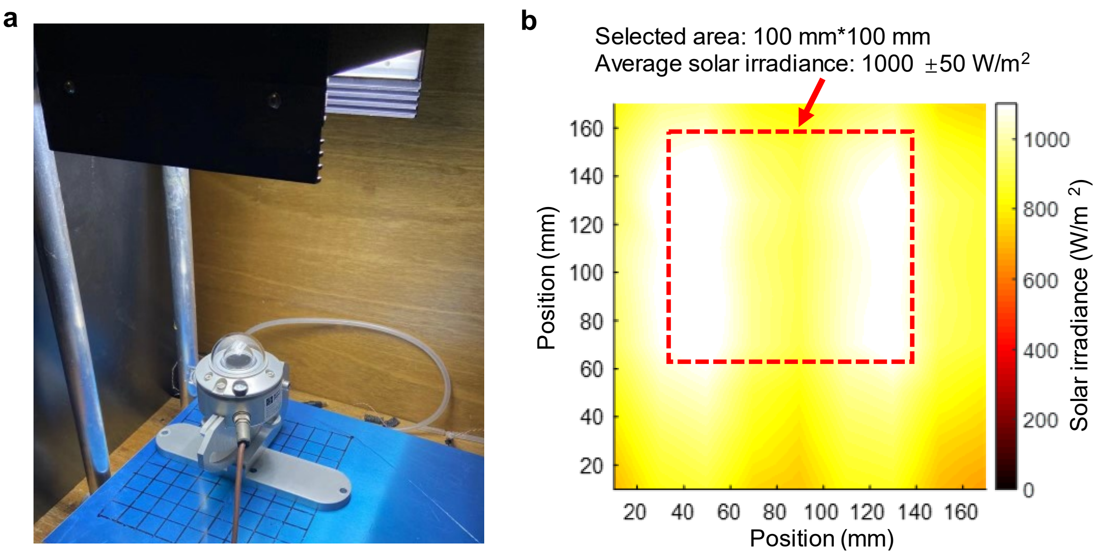

Supplementary Figure 3. Solar irradiance spatial distribution. a, Photograph of the illumination area of the solar simulator. The effective illumination window of the solar simulator is around 160 × 160 mm^2. The solar irradiance varies in both the horizontal and vertical directions. It is essential to calibrate the solar irradiance distribution and to select the most suitable area for testing. A first-class pyranometer was used to test the solar irradiance distribution. **b,** Solar irradiance distribution and selected area for the testing. A 100 × 100 mm^2 area marked with red dash lines was finally selected as the area for the testing. The average solar irradiance in the selected area is 1000 ± 50 W/m^2.

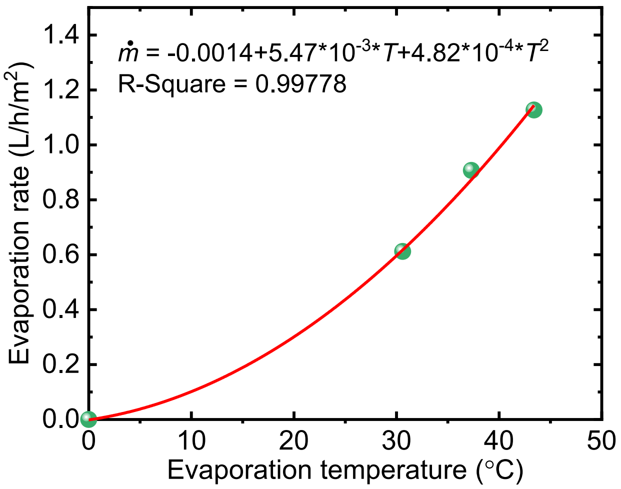

Supplementary Figure 4. Evaporation rate achieved in the PV-leaf as a function of the evaporation temperature. The evaporation rate within the PV-leaf was tested under the solar simulator, at an ambient temperature of 33.5 °C and relative humidity of 10%. The temperature of the PV-leaf was adjusted by changing the solar irradiance. The evaporation rate is a function of the ambient conditions (temperature, humidity, pressure, wind speed), evaporation surface (material and structure), and evaporation temperature[1,3]. Therefore, for given ambient conditions and evaporation surface, the evaporation rate is a function of the interface evaporation temperature. In the limit of the interface temperature being at 0 °C, the theoretical evaporation rate is close to zero. The evaporation curve indicates the transpiration performance of the PV-leaf and was also used as an input for the numerical modelling in this study.

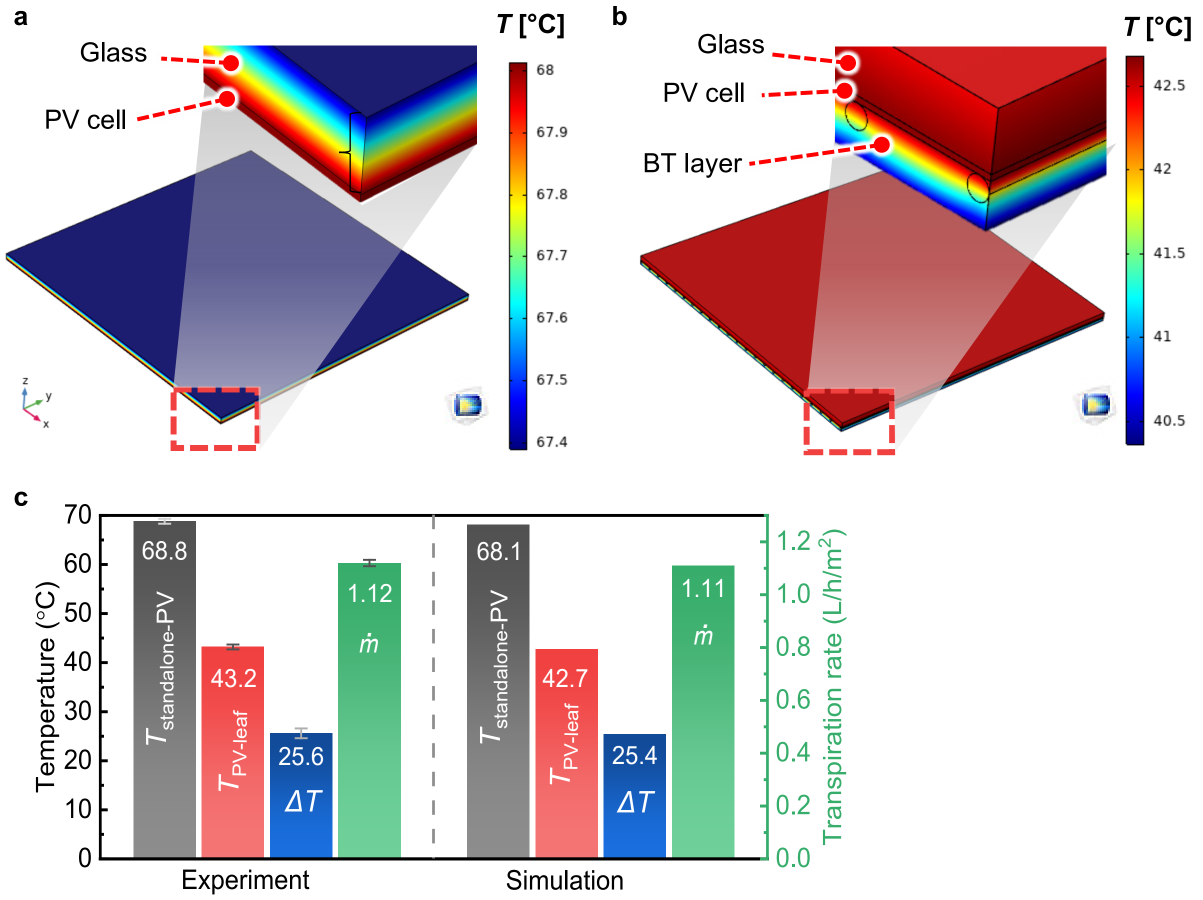

Supplementary Figure 5. COMSOL simulation and validation. a, Temperature distributions in the standalone PV panel (G = 1000 W/m^2, T_a = 33.5 °C, *Hum* = 10%). The PV cell experiences a high temperature due to the significant waste heat and poor natural heat dissipation. **b,** Temperature distributions in the PV-leaf (G = 1000 W/m^2, T_a = 33.5 °C, *Hum* = 10%). The evaporation curve in Supplementary Figure 3 is used to map the evaporation rate. The stainless-steel mesh and the structured surface of the BT layer are not considered in the model. The PV cell, which is at 42.7 °C, remains the hottest area in the PV-leaf but is significantly cooler than the cell in the standalone PV cell. The surface of the BT layer is ~2 °C lower than the PV cell due to the thermal resistance of the BT layer, which can be further reduced by suspending high-heat-conduction materials such as metal powders or carbon nanotubes[4,5]. **c,** Model validation with indoor test data. The simulated temperatures of the standalone PV cell and PV-leaf are close to the experimental results, with relative errors below 5%. The simulated transpiration rate of the PV-leaf is 1.11 L/h/m^2, which is close to the experimental result of 1.12 L/h/m^2.

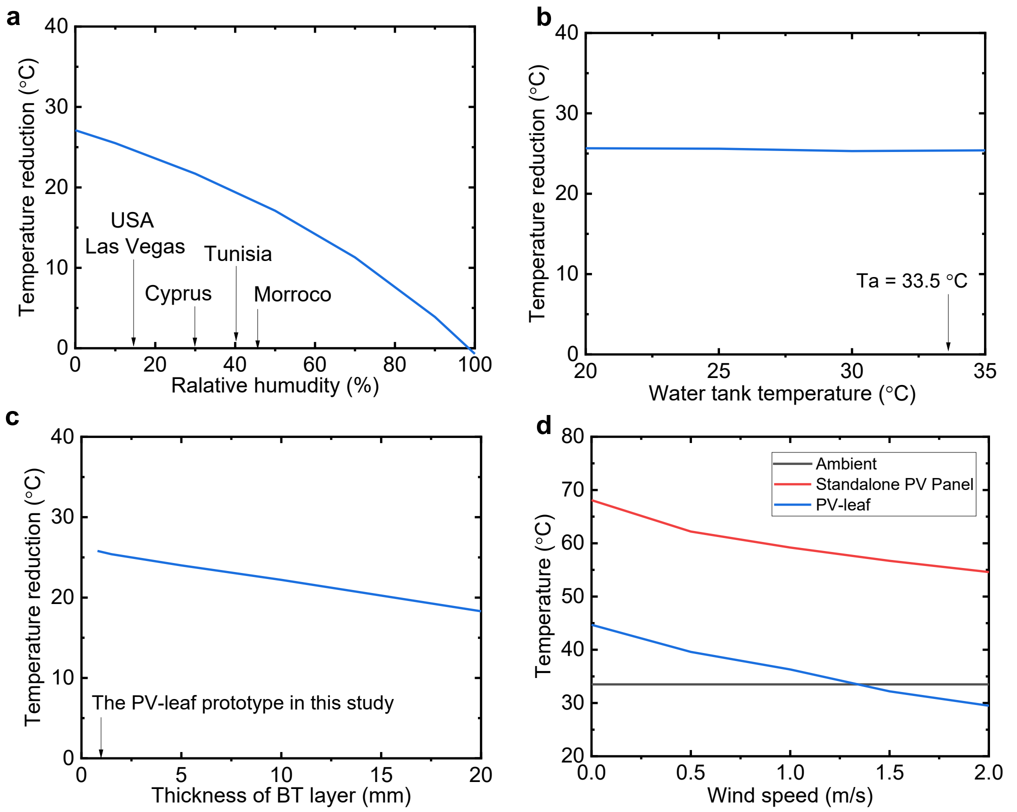

Supplementary Figure 6. Influence of key parameters on PV-leaf transpiration performance. A validated 3-D COMSOL model was used to investigate the effects of humidity, the water tank temperature, BT layer thickness and wind speed on PV-leaf performance. **a,** Impact of relative humidity on the cooling performance. According to the experimental results in Supplementary Ref. 7, the evaporation rate decreases linearly as the relative humidity increases. The evaporation rate is theoretically zero, and there is no cooling power, when the relative humidity is 100%. The temperature reduction (i.e., temperature difference between the PV-leaf and the standalone PV cell) decreases as the relative humidity increases. **b,** Impact of coolant water supplying temperature (i.e., water-tank temperature) on the cooling performance. The water supplying temperature (in the range 20-35 °C) only has a slight influence on the temperature reduction. The latent heat of evaporation of water is over an order of magnitude larger than the sensible heat of water. Therefore, a decrease in the coolant water temperature only has a slight impact on the cooling performance of the PV-leaf. **c,** Impact of BT layer thickness on the cooling performance. Transpiration cooling occurs on the bottom surface of the BT layer. The thermal resistance between the PV cell and the BT layer's bottom surface increases as the BT layer thickness increases, so a thinner BT layer results in a lower PV cell temperature and better cooling performance. Of note is that the BT layer thickness should be larger than the diameter of the bamboo fibre bundles (i.e., >0.8 mm) for water transportation. The thickness of the BT layer was thus finally selected to be 1 mm in the prototype. **d**, Impact of wind speed on the PV-leaf temperature. In the model, the relationship between the convective heat transfer coefficient and wind speed was taken from Supplementary Ref. 8. The evaporation rate increases significantly at higher wind speeds. The influence of the wind speed on the evaporation rate is considered in Supplementary Ref. 9 (*i.e.*, the evaporation rate increases by 80% when the wind speed increases from 0 m/s to 1 m/s and by 2.7 times when it increases from 0 m/s to 2 m/s). Simulation results show that the temperatures of the PV panel and PV-leaf both decrease as the wind speed increases. The PV-leaf temperature can even be lower than the ambient temperature when the wind speed is higher than the critical wind speed of ~1.5 m/s.

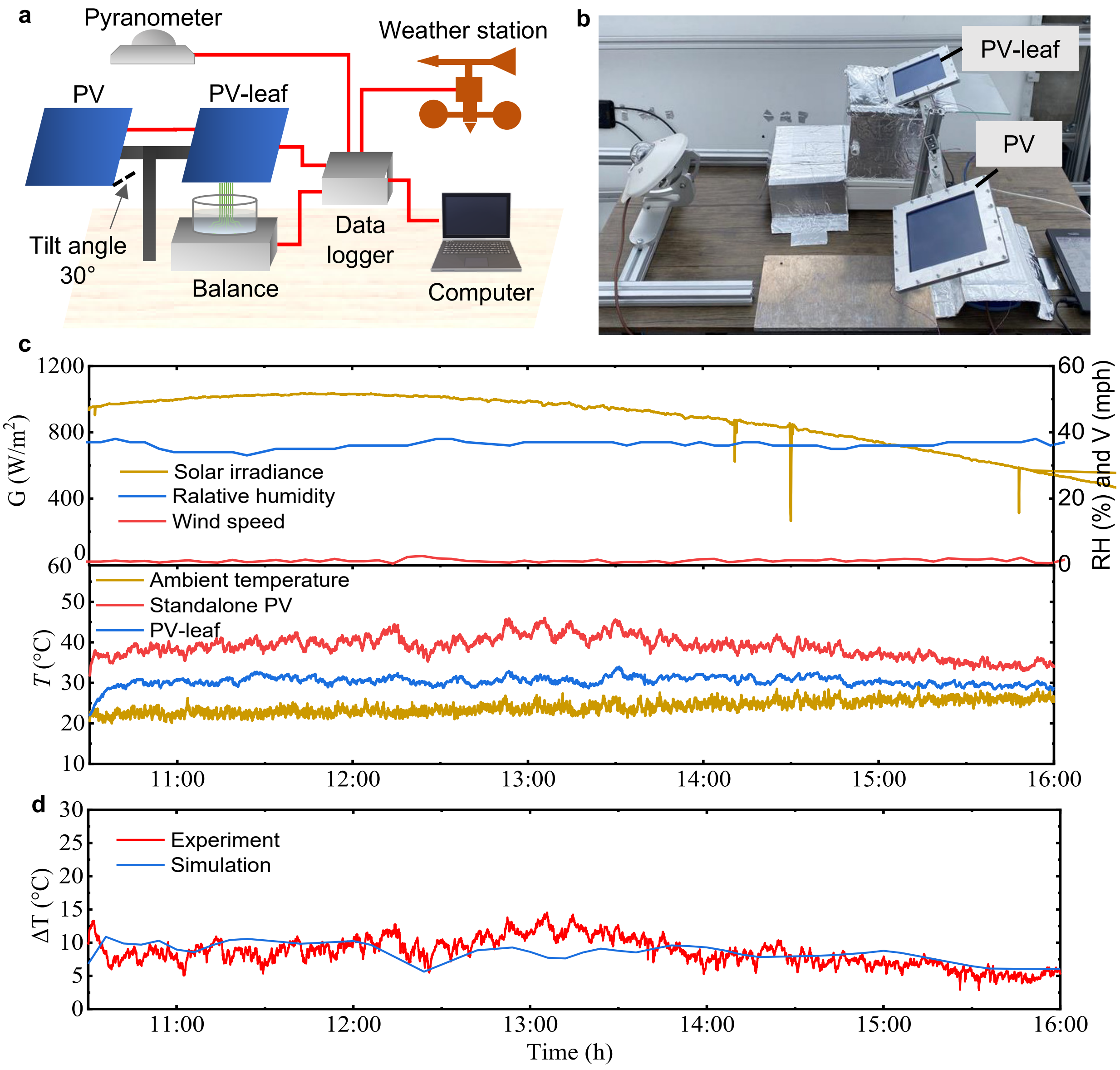

Supplementary Figure 7. Outdoor testing of the PV-leaf and standalone PV panel. a, Diagram of the outdoor testing platform. Both the PV-leaf and the standalone PV cell were oriented southwards and titled 30°. A pyranometer and a weather station were used to measure the solar irradiance, wind speed, relative humidity and ambient temperature. Both the PV-leaf and the standalone PV cell were tested at electricity-off conditions. **b,** Photograph of the outdoor testing platform located on the roof of the Roderic Hill Building in the South Kensington Campus of Imperial College London (51° 29' 58.11" N 0° 10' 41.7648" W). The electronic balance was protected by a wind and sun shield. **c,** Outdoor test results. The experiments were conducted under a clear sky on 22 June 2022. The solar irradiance peaked at 12:08 with a value of 1036 W/m^2. The average relative humidity and wind speed during 10:30-16:00 were 38% and 0.5 m/s. The temperature of the PV-leaf reached 30.9 °C at 12:30, which was 6.8 °C higher than the ambient temperature. The standalone PV cell temperature changed with the solar irradiance and ambient temperature, while the PV-leaf temperature was significantly less variable, staying at around 30 °C from morning to afternoon. The PV-leaf was able to passively control the cooling effect in a real outdoor environment and had a stable diurnal (whole day) cooling performance. **d,** Reduction in the PV-leaf temperature relative to the standalone PV cell. The cooling performance of the PV-leaf was more noticeable at higher solar irradiances. The temperature

reduction was around 6.8 °C at 10:30, and increased to 10.1 °C at 12:00. The maximum and average temperature reduction during 10:30-16:00 were 14.5 °C and 8.5 °C, resulting in a relative PV efficiency improvement of 6.8% and 4.1%. The PV-leaf will have a better cooling performance when the prototype is located at dryer and hotter regions, such as in Cyprus. The maximum temperature reduction achieved in the indoor experiments was 18.5 °C when the laboratory ambient temperature was 24.1 °C and with a relative humidity of 10%. Of note is that the maximum temperature reduction in the outdoor experiments (14.5 °C) was lower than that in the indoor experiments (18.5 °C), due to the difference in the relative humidity. Transient simulations in COMSOL agree well with these experimental results, also further validating the model.

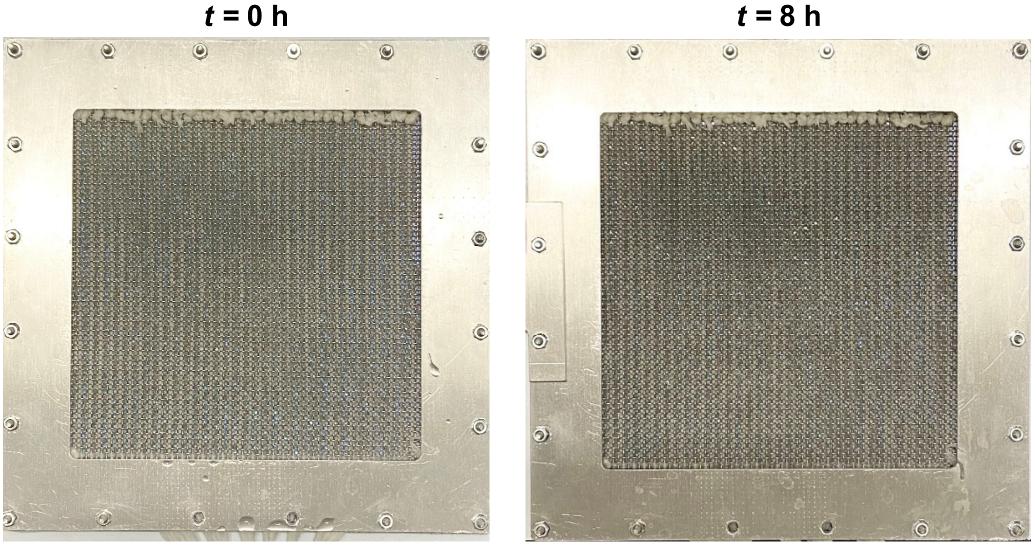

Supplementary Figure 8. Photograph of the bottom of the PV-leaf transpiration structure after 8 hours of continuous operation under peak solar irradiance (*G* = 1000 W/m^2). No obvious salt crystallization was observed on the outer surface of the mesh after 8 h of continuous testing. To ensure the sustainable operation of the PV-leaf over a longer time scale, low-cost regular maintenance may be required to remove any accumulated salt more effectively from the BT layer by flushing seawater, as suggested in Supplementary Ref. 10. Of note is that the effect of scaling formed by magnesium species in real seawater on PV-leaf performance also requires additional attention (Supplementary Ref. 11).

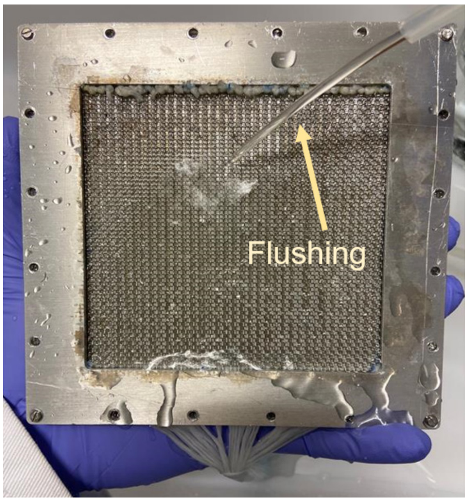

Supplementary Figure 9. Low-cost regular maintenance by using seawater flushing. Low-cost regular maintenance, such as seawater flushing, is required to remove the accumulated salt in the BT layer, which was also suggested in Supplementary Ref. 10.

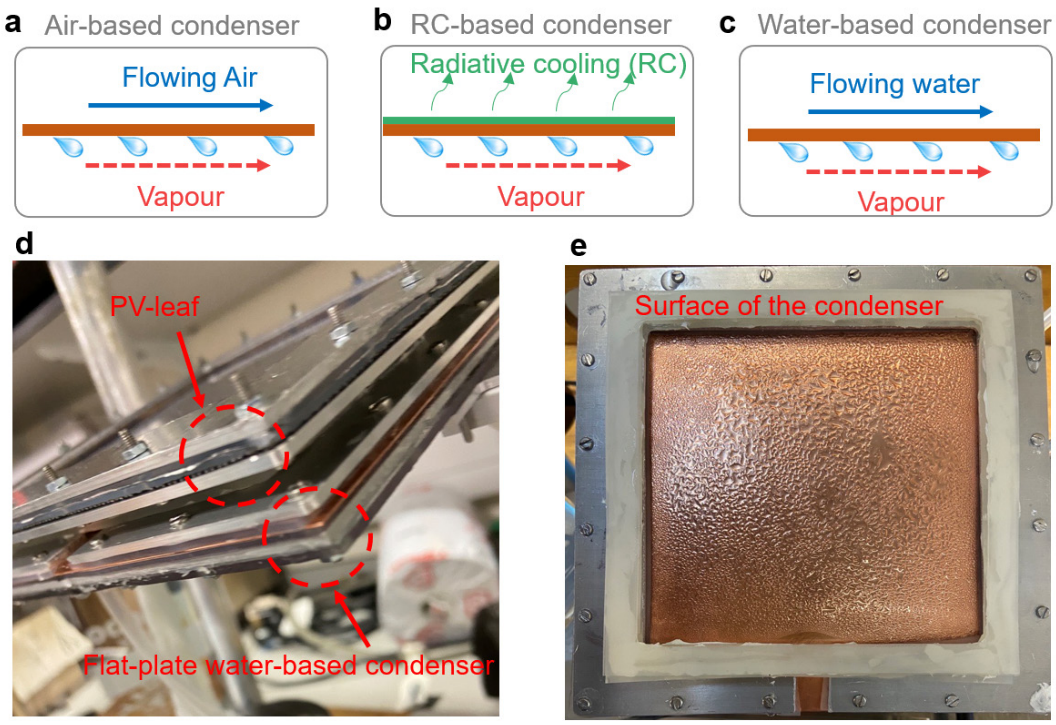

Supplementary Figure 10. Different types of condensers. a, Air-based condenser removes heat by employing an ambient-temperature air flow to condense the vapour. **b,** Radiative-cooling-based condenser, as proposed in Supplementary Ref. 12, emits heat to a cold space. **c,** Water-based condenser uses flowing cold water as the coolant to remove heat with a higher heat transfer coefficient compared to an air-based condenser. **d,** A tailored flat-plate water-based condenser was used to condense the vapour generated by the PV-TD-leaf. **e,** A water film is generated on the condensation surface. The desalinated water is then collected to measure its salinity.

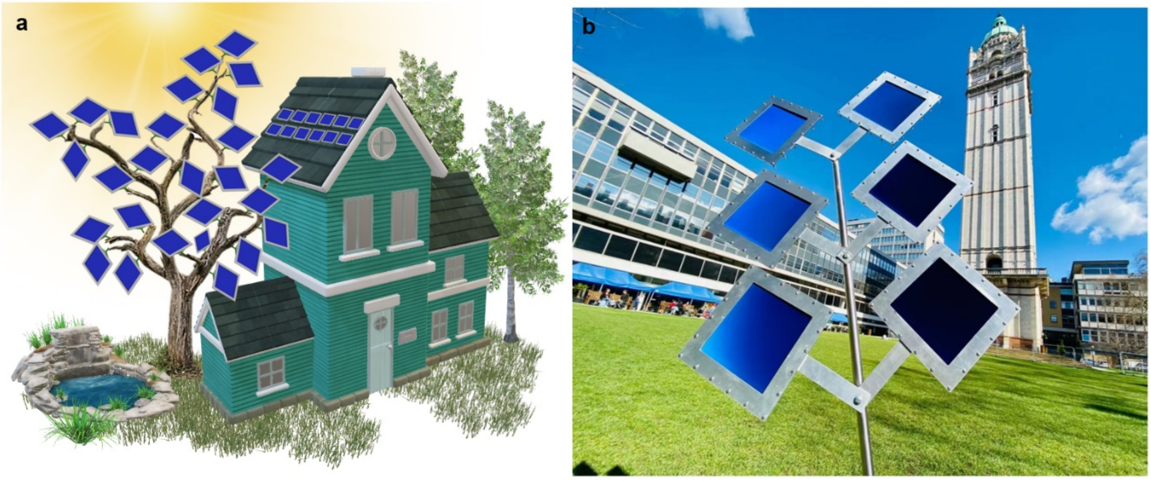

Supplementary Figure 11. Concept of a large-scale PV-tree. a, Perspective drawing of a PV-tree for domestic applications. Such a PV-tree presents an option for installing multiple layers of PV-leaves in a way that maximizes the total useful energy output from a limited available area. The working fluid is transported from the root to the PV-leaves for cooling and desalination (or water cleaning). Alternatively, PV-leaves can also be mounted on roofs in a traditional way. Based on the performance of a single PV-leaf, a 50 m^2 total area of PV-leaves is estimated to be able to meet both the electricity and freshwater demands of a typical house of 4 inhabitants. **b,** Photograph of a PV-tree branch with PV-leaves. A convenient way to scale up the system is to connect several PV-leaves together. For example, we fabricated a PV-tree branch to illustrate the possibility of such Lego-like reconfigurations for larger-scale systems. Here, six PV-leaves are connected to a common supply tube through which water flows continuously to all PV-leaves driven by capillarity. (N.B.: The present article mainly focuses on proposing the concept and presenting the design of a single PV-leaf, along with preliminarily investigations of its performance. The investigation of larger-scale systems is beyond the scope of the article, but is part of ongoing work, which requires a focus on system optimization. The purpose of this figure is to illustrate, conceptually, the potential of such scalable PV-leaf systems.)

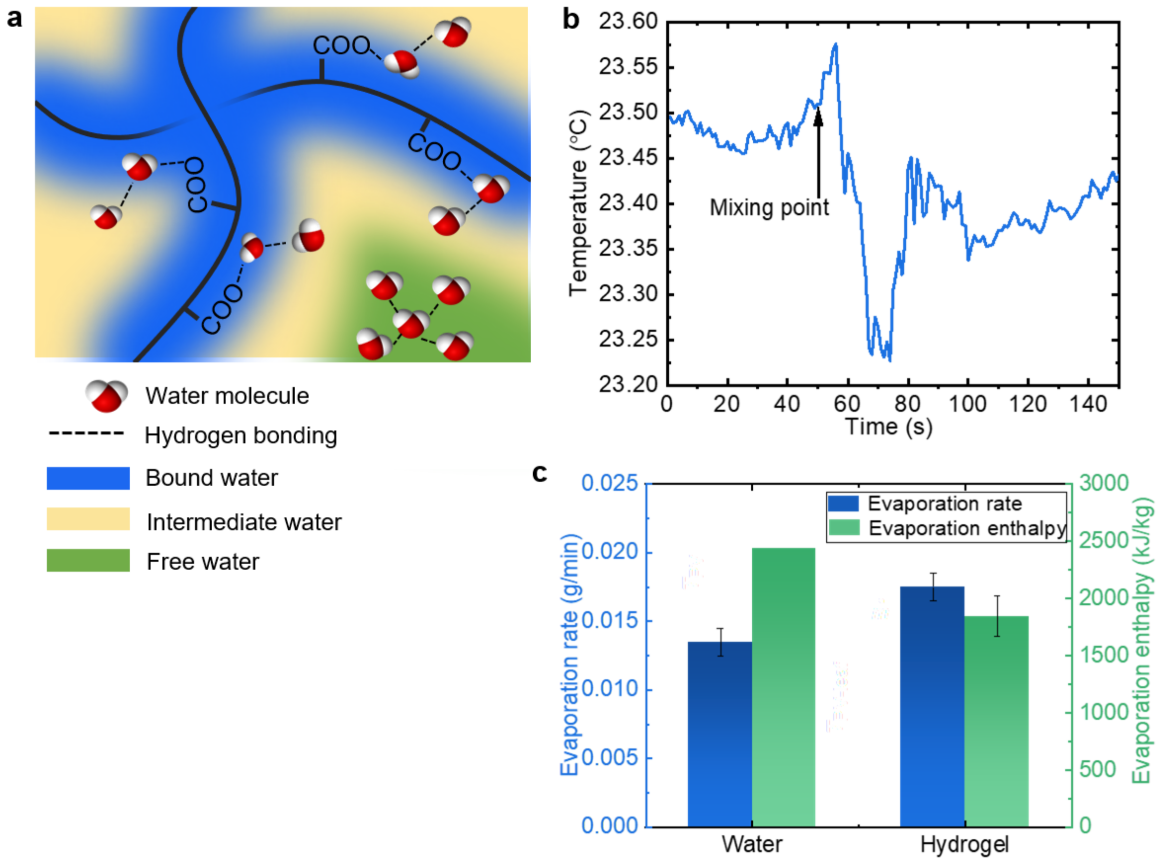

Supplementary Figure 12. Characterization of evaporation enthalpy of PAAK hydrogel. **a**, Schematic of water-polymer bonding (bound water), weakened water-water bonding (intermediate water), and normal water-water bonding (free water) in a hydratable polymer network. In bound water (the blue area in the figure), water molecules are strongly bonded to –COO− groups in the PAAK polymer mesh. The free water (green area) in the polymer network has the same evaporation enthalpy as that of bulk water. In between the bound water and free water, intermediate water (yellow area) exists and is weakly bonded to polymer chains and adjacent water molecules [13-15]. **b**, Temperature changes when mixing PAAK with water. Dry PAAK particles and water were sealed and placed in the same laboratory environment for a sufficient time until they reached the same temperature, as measured by a thin (0.2 mm), high-precision (T-type, ±0.1 K) thermocouple. A sudden temperature reduction was observed, as expected by an endothermic process, when mixing in a sealed tank (no evaporation impact). The temperature of the mixture then gradually increased back to the initial temperature by absorbing heat from the ambient. This endothermic phenomenon also agrees with the experimental observations of Di Maggio et al. [16]. **c**, Evaporation rates and calculated equivalent evaporation enthalpy values of water and of the hydrogel. The surface of the hydrogel was carefully cut, pressed and shaped to be as flat as possible, thus making the evaporation surface areas of the bulk hydrogel and the bulk water as close as possible. Both the hydrogel and water samples were sealed and placed in the same laboratory environment with sufficient time before starting the characterization experiments. The evaporation rates for the hydrogel and water samples were calculated based on the weight reduction gradients near the starting time of the experiment, at which time the samples had been measured to have the same surface temperature. The characterization experiment for the hydrogel was based on the widely-used methodology proposed by Yu's group [13,17,18], which is based on the assumption that the bulk hydrogel and bulk water have identical heat inputs from the environment. Error bars are included representing the measurement uncertainty.

Supplementary Table 1. Capital costs of conventional PV panel and PV-leaf[19].

	Item	Price ($\$/m^2$)	Percentage
**Conventional PV panel**	PV panel	55	-
**PV-leaf**	PV panel	55	-
	BT layer (hydrogel)	0.1	2% of the PV panel
	BT layer (fibre bundle)	0.3	
	Supporting mesh	0.3	
	Piping	0.4	

All prices in the table are based on the available bulk pricing of materials[13]. A 1-m^2 PV-leaf requires around 300 m (or 85 g) of bamboo fibre bundles and 25 g of potassium polyacrylate particles. The prices of bamboo fibre and potassium polyacrylate are around 3-3.5 \$/kg and 2.5-3.5 \$/kg. Thus, the costs of the bamboo fibre bundle and the potassium polyacrylate are around 0.3 $\$/m^2$ and 0.1 $\$/m^2$. In practical applications, a polyethylene mesh can be employed as a supporting mesh in the PV-leaf, which is low-cost, light and durable. The price of the polyethylene mesh is around 0.3 $\$/m^2$. Polyethylene pipes with 10-mm diameter (around 0.4 \$/m) are assumed to be used to supply water. Assuming 1-m^2 PV-leaf requires about 1 m of polyethylene pipe, the cost of the pipe is 0.4 \$. The price of a high-performance monocrystalline PV panel is around 55 $\$/m^2$. The capital cost of the additional components (hydrogel, fibre bundle, supporting mesh and piping) is only ~2% of the price of commercial PV panels.

Supplementary References

1. I-V Characterization of Photovoltaic Cells and Panels Using the Keithley 2450 or 2460 SourceMeter. *TEKTRONIX Keithley*, https://www.tek.com/en/documents/application-note/i-v-characterization-photovoltaic-cells-and-panels-using-keithley-2450-or (2022).
2. Jones, F. E. Evaporation of water with emphasis on applications and measurements (*CRC Press*, 2018).
3. Sartori, E. A critical review on equations employed for the calculation of the evaporation rate from free water surfaces. *Sol. Energy* **68**, 77-89 (2020).
4. Chung, D. D. L. Materials for thermal conduction. *Appl. Therm. Eng.* **21**, 1593-1605 (2001).
5. Avery, A. D., Zhou, B. H., Lee, J., Lee, E. S., Miller, E. M., Ihly, R., ... & Ferguson, A. J. Tailored semiconducting carbon nanotube networks with enhanced thermoelectric properties. *Nat. Energy* **1**, 16033 (2016).
6. Photovoltaic Geographical Information System. *European Commission*, https://ec.europa.eu/jrc/en/PVGIS/tools/daily-radiation (2022).
7. Hisatake, K., Fukuda, M., Kimura, J., Maeda, M., & Fukuda, Y. Experimental and theoretical study of evaporation of water in a vessel. *J. Appl. Phys.* **77**, 6664-6674 (1995).
8. Kumar, S., & Mullick, S. C. Wind heat transfer coefficient in solar collectors in outdoor conditions. *Sol. Energy* **84**, 956-963 (2010).
9. Liu, F., Zhao, B., Wu, W., Yang, H., Ning, Y., Lai, Y., & Bradley, R. Low cost, robust, environmentally friendly geopolymer–mesoporous carbon composites for efficient solar powered steam generation. *Adv. Funct. Mater.* **28**, 1803266 (2018).
10. Singh, S. C., ElKabbash, M., Li, Z., Li, X., Regmi, B., Madsen, M., ... & Guo, C. Solar-trackable super-wicking black metal panel for photothermal water sanitation. *Nat. Sustain.* **3**, 938-946 (2020).
11. Zhang, C., Shi, Y., Shi, L., Li, H., Li, R., Hong, S., ... & Wang, P. Designing a next generation solar crystallizer for real seawater brine treatment with zero liquid discharge. *Nat. Commun.* **12**, 998 (2021).
12. Zhou, M., Song, H., Xu, X., Shahsafi, A., Qu, Y., Xia, Z., ... & Yu, Z. Vapor condensation with daytime radiative cooling. *Proc. Natl. Acad. Sci.* **118**, 14 (2021).
13. Zhou, X., Zhao, F., Guo, Y., Zhang, Y., & Yu, G. A hydrogel-based antifouling solar evaporator for highly efficient water desalination. *Energy Environ. Sci.* **11**, 1985-1992 (2018).
14. Lei, C., Guan, W., Guo, Y., Shi, W., Wang, Y., Johnston, K. P., & Yu, G. Polyzwitterionic hydrogels for highly efficient high salinity solar desalination. *Angew. Chem.* **61**, e202208487 (2022).
15. Chen, G. Thermodynamics of hydrogels for applications in atmospheric water harvesting, evaporation, and desalination. *Phys. Chem. Chem. Phys.* **24**, 12329-12345 (2022).
16. Di Maggio, R., Dirè, S., Callone, E., Bergamonti, L., Lottici, P. P., Albatici, R., ... & Ataollahi, N. Super-adsorbent polyacrylate under swelling in water for passive solar control of building envelope. *SN Appl. Sci.* **2**, 45 (2020).
17. Guo, Y., Lu, H., Zhao, F., Zhou, X., Shi, W., & Yu, G. Biomass ‐ derived hybrid hydrogel evaporators for cost ‐ effective solar water purification. *Adv. Mater.* **32**, 1907061 (2020).
18. Zhou, X., Zhao, F., Guo, Y., Rosenberger, B., & Yu, G. Architecting highly hydratable polymer networks to tune the water state for solar water purification. *Sci. Adv.* **5**, eaaw5484 (2019).
19. ALIBABA online wholesale suppliers, https://www.alibaba.com (2021).
