# Supplementary material for: High-efficiency bio-inspired hybrid multi-generation photovoltaic leaf
Source: Nat Commun. 2023 Jun 8;14:3344. doi: 10.1038/s41467-023-38984-7 (PMC10250451; doi:10.1038/s41467-023-38984-7)
Supplement: Supplementary file 2 — Solar Cells Reporting Summary [file 41467_2023_38984_MOESM2_ESM.pdf]

nature research

Corresponding author(s): Gan Huang; Christos N. Markides

Solar Cells Reporting Summary

Nature Research wishes to improve the reproducibility of the work that we publish. This form is intended for publication with all accepted papers reporting the characterization of photovoltaic devices and provides structure for consistency and transparency in reporting. Some list items might not apply to an individual manuscript, but all fields must be completed for clarity.

For further information on Nature Research policies, including our data availability policy, see Authors & Referees.

Experimental design

Please check: are the following details reported in the manuscript?

1. Dimensions

Item	Yes	No	Details
Area of the tested solar cells	[X]	[ ]	Method section
Method used to determine the device area	[X]	[ ]	Method section

2. Current-voltage characterization

Item	Yes	No	Details
Current density-voltage (J-V) plots in both forward and backward direction	[X]	[ ]	Figure 2
Voltage scan conditions<br>*For instance: scan direction, speed, dwell times*	[X]	[ ]	Figure 2
Test environment<br>*For instance: characterization temperature, in air or in glove box*	[X]	[ ]	Method section
Protocol for preconditioning of the device before its characterization	[ ]	[X]	the solar cell in this study is a commercially available product.
Stability of the J-V characteristic<br>*Verified with time evolution of the maximum power point or with the photocurrent at maximum power point; see ref. 7 for details.*	[ ]	[X]	the solar cell in this study is a commercially available product.

3. Hysteresis or any other unusual behaviour

Item	Yes	No	Details
Description of the unusual behaviour observed during the characterization	[ ]	[X]	the solar cell in this study is a commercially available product.
Related experimental data	[ ]	[X]	the solar cell in this study is a commercially available product.

4. Efficiency

Item	Yes	No	Details
External quantum efficiency (EQE) or incident photons to current efficiency (IPCE)	[ ]	[X]	the solar cell in this study is a commercially available product.
A comparison between the integrated response under the standard reference spectrum and the response measure under the simulator	[ ]	[X]	the solar cell in this study is a commercially available product.
For tandem solar cells, the bias illumination and bias voltage used for each subcell	[ ]	[X]	the solar cell in this study is a commercially available product.

5. Calibration

Item	Yes	No	Details
Light source and reference cell or sensor used for the characterization	[X]	[ ]	Supplementary figure 3
Confirmation that the reference cell was calibrated and certified	[X]	[ ]	the solar cell in this study is a commercially available product.

Calculation of spectral mismatch between the reference cell and the devices under test

- [ ] Yes
- [x] No

the solar cell in this study is a commercially available product.

6. Mask/aperture

Size of the mask/aperture used during testing

- [ ] Yes
- [x] No

N/A

Variation of the measured short-circuit current density with the mask/aperture area

- [ ] Yes
- [x] No

N/A

7. Performance certification

Identity of the independent certification laboratory that confirmed the photovoltaic performance

- [ ] Yes
- [x] No

the solar cell in this study is a commercially available product.

A copy of any certificate(s)
Provide in Supplementary Information

- [ ] Yes
- [x] No

the solar cell in this study is a commercially available product.

8. Statistics

Number of solar cells tested

- [x] Yes
- [ ] No

Method section

Statistical analysis of the device performance

- [x] Yes
- [ ] No

Results section

9. Long-term stability analysis

Type of analysis, bias conditions and environmental conditions
For instance: illumination type, temperature, atmosphere humidity, encapsulation method, preconditioning temperature

- [x] Yes
- [ ] No

Figure 5
